# Forecasting demand for maternal influenza immunization in low- and lower-middle-income countries

**DOI:** 10.1371/journal.pone.0199470

**Published:** 2018-06-22

**Authors:** Frédéric Debellut, Nathaniel Hendrix, Justin R. Ortiz, Philipp Lambach, Kathleen M. Neuzil, Niranjan Bhat, Clint Pecenka

**Affiliations:** 1 Center for Vaccine Innovation and Access, PATH, Geneva, Switzerland; 2 Center for Vaccine Innovation and Access, PATH, Seattle, Washington, United States of America; 3 Center for Vaccine Development, University of Maryland, Baltimore, Maryland, United States of America; 4 Initiative for Vaccine Research, World Health Organization, Geneva, Switzerland; Instituto Butantan, BRAZIL

## Abstract

Immunization of pregnant women against seasonal influenza remains limited in low- and lower-middle-income countries despite being recommended by the World Health Organization (WHO). The WHO/PATH Maternal Influenza Immunization Project was created to identify and address obstacles to delivering influenza vaccines to pregnant women in low resource setting. To gain a better understanding of potential demand from this target group, we developed a model simulating pregnant women populations eligible for vaccination during antenatal care (ANC) services in all low- and lower-middle-income countries. We assessed potential vaccine demand in the context of both seasonal and year-round vaccination strategies and identified the ways that immunization programs may be affected by availability gaps in supply linked to current vaccine production cycles and shelf life duration. Results of our analysis, which includes 54 eligible countries in 2015 for New Vaccine Support from Gavi, the Vaccine Alliance, suggest the demand for influenza vaccines could be 7.7 to 16.0 million doses in 2020, and 27.0 to 61.7 million doses by 2029. If current trends in production capacity and actual production of seasonal influenza vaccines were to continue, global vaccine supply would be sufficient to meet this additional demand—although a majority of countries would face implementation issues linked to timing of supply.

## Introduction

The World Health Organization (WHO) recommends that countries expanding or initiating influenza vaccine programs prioritize pregnant women for vaccine receipt [1]. One dose of influenza vaccine can prevent influenza illness in two high risk groups: pregnant women and their newborn children [2–4]. Despite the evidence of vaccine safety and efficacy [5–8], implementation of maternal influenza immunization programs has been limited in low- and lower-middle-income countries [9;10]. Challenges include vaccine availability, limitations in delivery logistics and cold chain capacity, timing of vaccination, anticipated coverage of immunization for pregnant women, vaccine acceptance, reliance on delivery platforms developed for children younger than 2 years of age or antenatal care, and the costs of vaccines and their delivery. These major factors need further investigation to facilitate influenza vaccine use in low resource settings.

The interplay of these issues is complex and makes planning for global implementation of maternal influenza programs challenging. Influenza vaccine composition is updated twice annually to produce Northern and Southern Hemisphere formulations [11]. Recommendations for each formulation come at different times—in February for the Northern Hemisphere formulation and in September for the Southern Hemisphere formulation. Vaccine expiration dates are chosen to align with temperate country influenza seasons and to minimize overlap regarding stock rotations [12]. As many tropical and subtropical countries have differing influenza virus circulation patterns compared to temperate countries, ensuring availability of vaccines can be challenging, leaving countries to choose between using outdated formulations; rushing to immunize before an expiration date; alternating between formulations when they are available; or not vaccinating a portion of the eligible population.

In 2013, the WHO/PATH Maternal Influenza Immunization Project was created to address obstacles to delivering influenza vaccines to pregnant women in low resource settings [13]. A key goal of this project was to gain a better understanding of vaccine supply needs and potential demand though the development of a demand forecasting model. The important role that organizations such as UNICEF and Gavi, the Vaccine Alliance (Gavi) play in vaccine procurement and financing, including Gavi’s stated goal of shaping vaccine markets, highlights the importance of these analyses [14]. In addition to informing Gavi and UNICEF about the potential financing and procurement implications of maternal influenza programs in low- and lower-middle-income countries, these analyses can be used to inform supply strategies for manufacturers to help define and estimate the size of possible future markets for influenza vaccine, which may contribute to a stable market.

This work builds upon the demand forecasting methods published in the literature and used to inform national and global policymakers [15–17]. It also extends prior work by examining a vaccine that is often delivered seasonally, adding nuance that is not typically considered in the demand forecasting literature. This article presents the model, describes methods used to develop it, provides estimates of potential demand for seasonal influenza vaccine generated by maternal influenza immunization programs in low- and lower-middle-income countries, and explores how these estimates may affect policy related to seasonal influenza vaccination.

## Materials and methods

### Model structure

**Fig 1:**
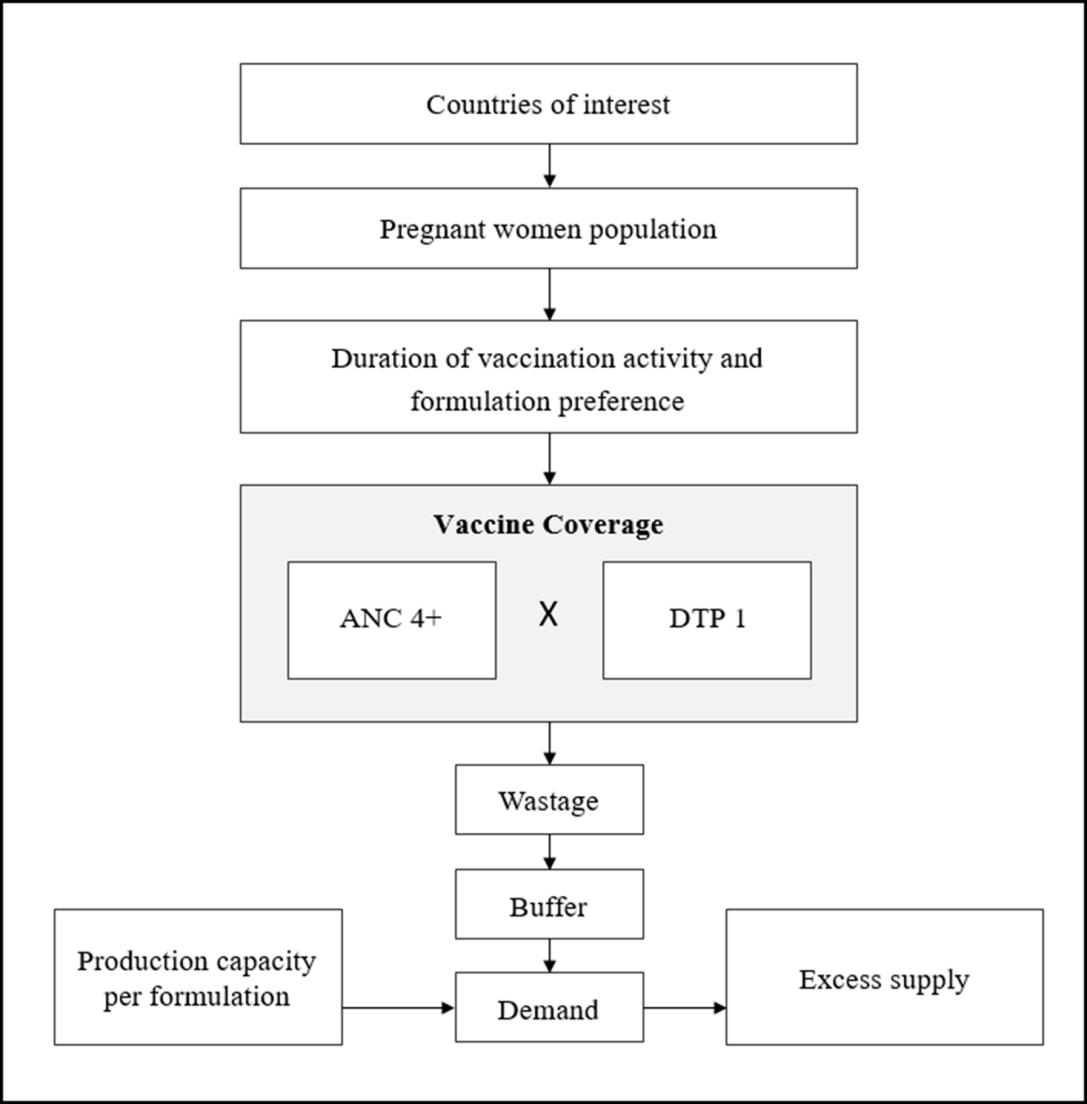
Model schematic (base case scenario).

Using a standard spreadsheet application (Excel, Microsoft Corp, Redmond, WA, US), we developed a model to estimate overall demand for influenza vaccines by pregnant women. The model simulates country-specific pregnant women populations in 80 low- and lower-middle-income countries for ten consecutive years, using projected data from 2020 to 2029. We assumed that none of the countries included in the analysis has an existing maternal influenza immunization program at the onset. A schematic of the model is displayed in Fig 1 with additional details on data inputs below.

### Model inputs

#### Countries and introduction dates

The model includes all 80 low- and lower-middle-income countries, filtered by income level [18] and further categorized by WHO region and eligibility to receive support from Gavi for new vaccine introduction as of December 2015 [19].

Each country generates annual vaccine demand from the time it initiates its maternal influenza immunization program. Countries were categorized as early, medium, or late adopters based on the time interval between vaccine availability and prior vaccine introductions (pneumococcal conjugate vaccine [PCV], rotavirus, and human papillomavirus [HPV] vaccines). Early adopters were assumed to start their maternal influenza program in 2020, medium adopters in 2022, and late adopters in 2025. This rather aggressive uptake assumption aims to evaluate maximum demand and potential issues around manufacturing capacity and cycles rather than developing a forecast of probable demand. The model also assumes that large countries will reach full coverage in a linear manner over three years, while smaller countries would achieve this in a single year. We considered a country to be large if its yearly birth cohort is greater than 2.5 million. Country groups and introduction dates are displayed in S1 Table.

#### Population and antenatal care attendance

We used the United Nations’ medium population projection [20] to estimate the number of live births per year for each country. We smoothed five year projected population estimates to avoid sharp changes between five year periods (methods described in S1 Fig). We estimated the number of pregnant women in each country by year by adding estimates of annual live births and stillbirths [21].

The model considers that influenza vaccines would be administered during antenatal care (ANC) visits, and a proportion of women who attend the recommended four visits will receive influenza vaccine during the second or third trimester. The number of pregnant women is then multiplied by the proportion that attend at least four ANC visits (ANC4+). Alternative scenarios examine the proportion of women attending at least one ANC visit (ANC1+). The use of ANC4+ and ANC1+ was mainly driven by global data availability but these inputs can be viewed as upper and lower bounds of the plausible target population [22].

To determine the probability a pregnant woman attending ANC receives the influenza vaccine, the number of pregnant women is multiplied by the coverage of the first dose of diphtheria-tetanus-pertussis (DTP1). While other proxies for coverage were considered, DTP1 was selected as a measure of country routine immunization performance and to reflect coverage of a single dose schedule vaccine [23].

#### Influenza seasonality and vaccine formulation

Influenza virus activity peaks at different times in different regions. Broadly, the temperate Northern Hemisphere tends to exhibit the highest levels of influenza activity from November to April, while influenza activity peaks during April to November in the temperate Southern Hemisphere. However, a limited number of countries exhibit a pattern of two annual peaks, and a few, mostly equatorial countries, exhibit influenza activity year-round [24–26].

Seasonal peaks of influenza virus infection formed the basis for assumptions about the delivery strategy in each country. For countries with one annual peak of influenza activity, we estimated the duration of influenza immunization campaigns by adding a preparatory period of two months to the number of months during which peak activity is seen [24–26]. Countries with two peaks or year-round influenza activity were assumed to immunize year-round.

Because of the lack of information on influenza virus circulation for some countries, it was necessary to extrapolate data from neighbouring countries. This is particularly true for several countries in the African region. When required, we used extrapolation to account for the circulation pattern of the closest country with data, giving preference to countries of similar latitude.

The choice of vaccine formulation depends on the months during which vaccination takes place. For countries with an official policy, we used their indicated preference for vaccine formulation [26–28]. For countries with no formal policy, we chose the formulation that would be available closest to the start of their vaccination activity. Countries that vaccinate year-round are assumed to use equal quantities of Northern and Southern Hemisphere vaccines.

Each country’s immunization strategy affects its demand for influenza vaccine. For example, in a country vaccinating during five months of the year, the population eligible for vaccination would be 5/12 (42%) of women who attend ANC annually.

#### Wastage and buffer stock

Demand estimates are further modified by the amount of vaccine wastage (accounting for doses that are lost) and the programmatic requirements for the maintenance of a buffer stock (accounting for doses held as a reserve to cover unforeseen shortages or demands).

We derived wastage rates from WHO studies of the effect of vial size and vaccine presentation on wastage. These studies estimate the wastage rate of liquid vaccine in ten-dose vials at 25 percent and in single dose vial at 5 percent [29]. The base case scenario assumes that countries would be using a single dose vial presentation.

The requirement for a buffer stock highlights complex policy and logistical issues. For most vaccines, it is recommended countries have an excess supply representing three months’ worth of vaccines—or 25 percent of their annual supply—which could then be incorporated into the supply for the following year. However, because the influenza vaccine’s formulation is re-evaluated each year, this efficiency would not be realized. The role of a buffer stock in an influenza vaccination program might be to allow the country to weather short-term interruptions in supply or sudden increases in demand—as might occur during a particularly severe influenza season. In the absence of any buffer stock policy for maternal influenza immunization but recognizing the increased expense of large annual buffer stocks for influenza vaccine, we used a 10 percent buffer in the base case scenario.

#### Vaccine supply

Resulting demand estimates were compared with global estimates of seasonal influenza vaccine supply generated by the WHO as part of the Global Action Plan for Influenza Vaccines (GAP). GAP has periodically surveyed manufacturers to assess how supply of seasonal and pandemic influenza vaccines is evolving over time. Surveys covered questions around manufacturers’ licensed seasonal or pandemic influenza vaccines, including information on vaccines’ composition, production processes, production location, number of doses produced during the last influenza season and number of doses that could be produced at maximum capacity. We used these surveys to recover data on production capacity and actual production [30–33].

## Results

### Base case scenario

We evaluated various scenarios to compare estimates of demand and available supply. The base case scenario examines demand in countries that were eligible to apply for New Vaccine Support (NVS) from Gavi in 2015 (a total of 54 countries) and applies the values described above for the model parameters (a summary of model inputs is displayed in S2 Table).

**Fig 2:**
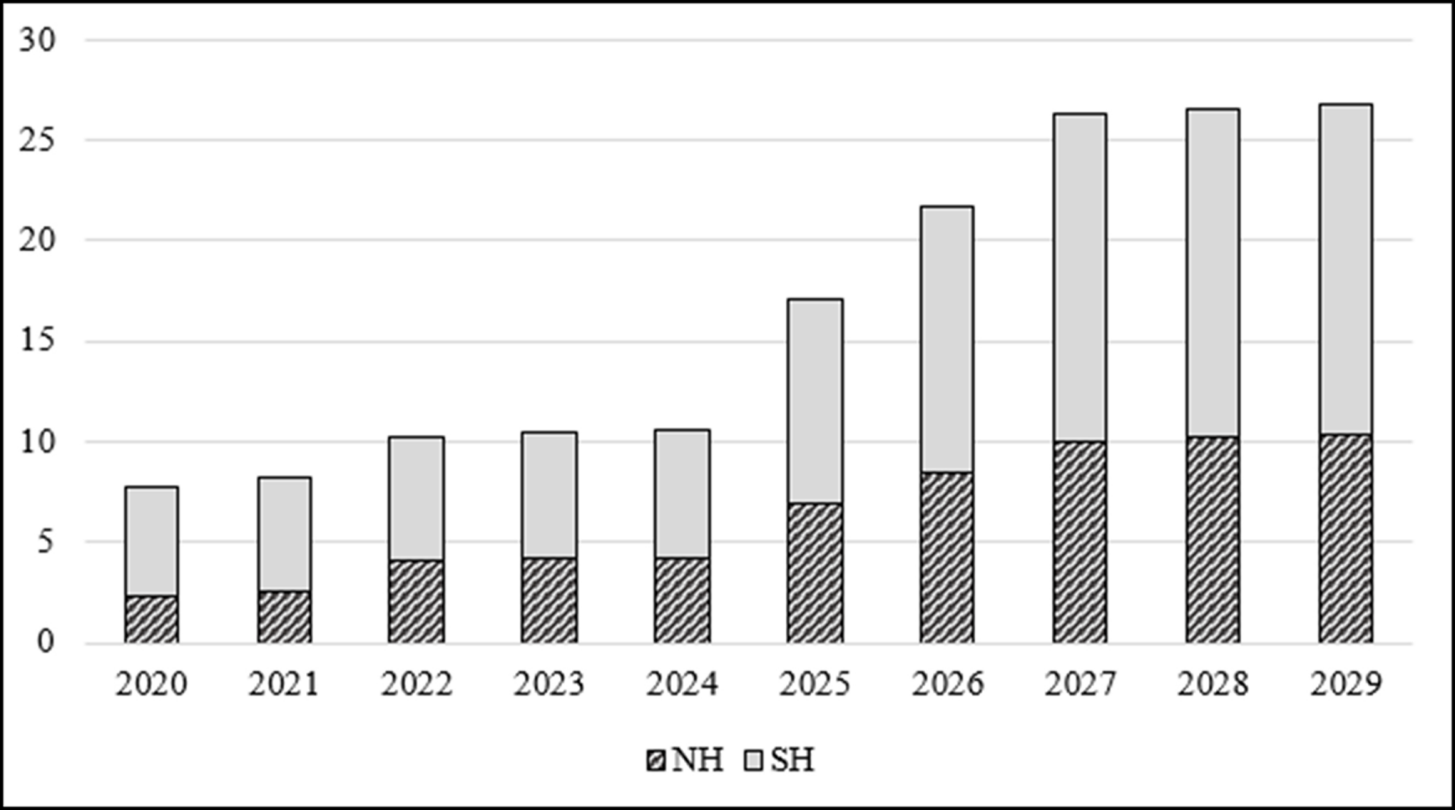
Demand estimates for the base case scenario (in millions of doses).

Demand estimates generated by the base case scenario are shown in Fig 2. The number of doses required for pregnant women in these countries begins at 7.7 million doses in 2020 and increases to nearly 27.0 million doses in 2029 as additional countries introduce the intervention and large countries expand coverage. This increase is highly influenced by assumptions around time of introduction, with a large number of countries starting their program in 2025, including most large countries (Nigeria, India, Bangladesh, DR Congo, and Pakistan). Therefore, the demand increase across the period is slow from 2020 to 2024 but accelerates sharply from 2025 to 2027. The share of Northern and Southern Hemisphere vaccine formulations fluctuates over time with the Southern Hemisphere formulation representing around two-thirds of the total demand in 2020 and decreasing slightly, to approximately 60 percent in 2029. The geographic distribution of demand in 2029 includes 63 percent of doses in the WHO African Region, 30 percent in the WHO South East Asian Region, and the remaining 7 percent shared among other WHO regions. Geographic distribution of demand estimates are displayed in S2 Fig.

In 2015, seasonal influenza vaccine annual production capacity was reported to be 1.46 billion doses, a level that remained stable from prior analyses [32–33]. Annual production capacity and actual supply of seasonal influenza vaccine therefore appear to be more than adequate to meet forecasted demand for maternal immunization in these 54 Gavi countries, although disparities between the Northern and Southern Hemisphere formulations need to be highlighted. While stratified estimates are not available from the last analysis, in 2011, the Northern Hemisphere formulation represented 75 percent of the total capacity.

### Alternative scenarios

**Fig 3:**
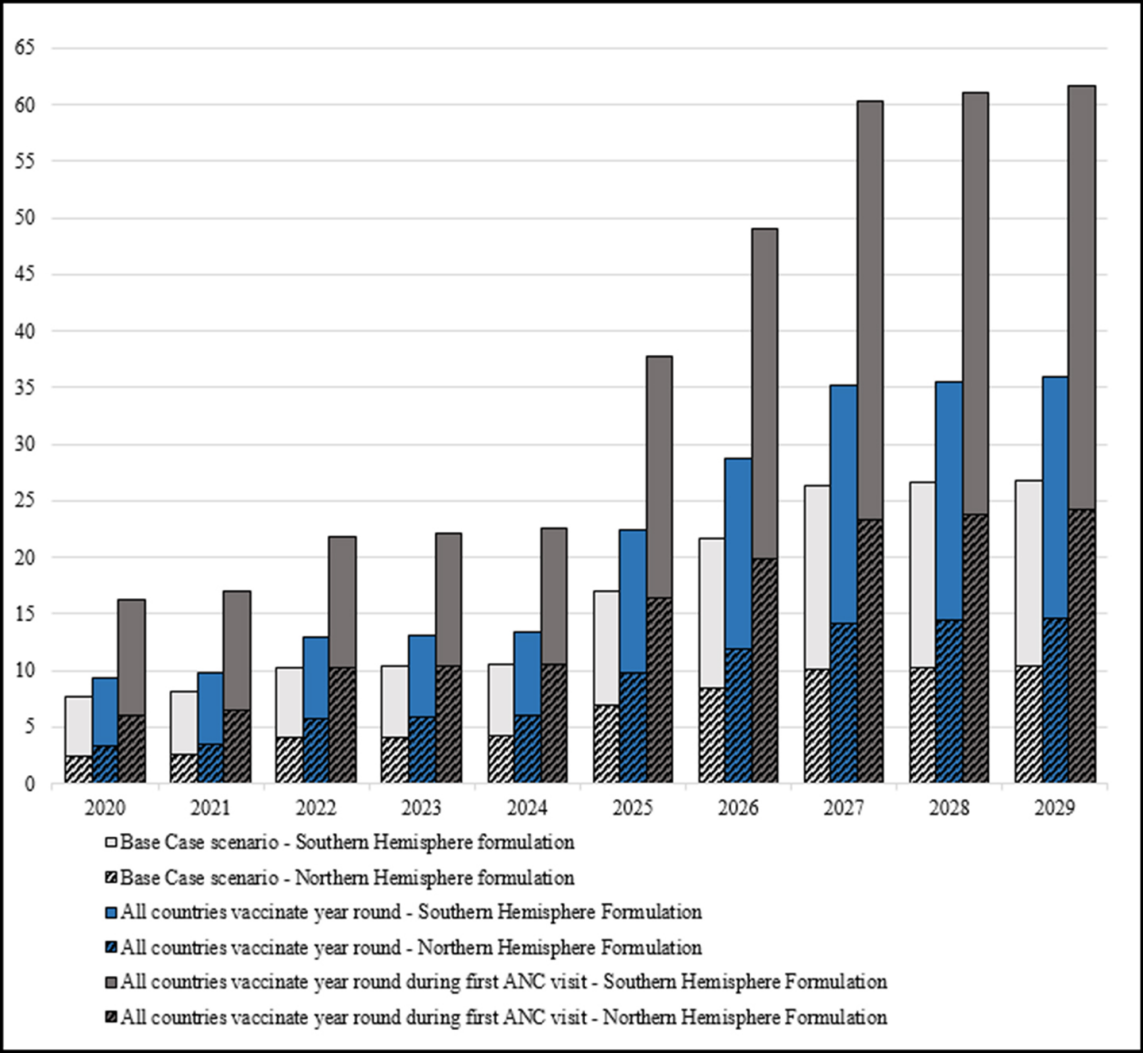
Comparison of demand estimates generated under base case scenario versus alternative scenarios (millions of doses).

We evaluated alternative scenarios in our model to confirm the robustness of our finding that supply capacity remains sufficient to meet overall demand. We considered two additional scenarios that incorporated assumptions considered to be viable options to deliver influenza vaccines and maximize disease prevention in the tropics and subtropics [12,34]. Because influenza vaccine can protect women through their pregnancy and maternal influenza immunization can protect infants for several months postpartum, an argument can be made for year-round maternal influenza immunization programs regardless of influenza seasonality. Therefore, for this alternative scenario, we assumed all 54 Gavi countries would vaccinate year-round. As an additional alternative scenario, we assumed influenza vaccine would be prioritized in antenatal care and given during the first healthcare encounter (using ANC1+ instead of ANC4+). Results from these two alternative scenarios and comparison with data from the base case are displayed in the Fig 3.

Despite a large increase in the number of doses compared to the base case—by 34 percent for the first alternative scenario and by 130 percent for the second (comparing year 2029)—available production capacity would still cover the additional demand generated for both formulations. This finding still holds in a final alternative analysis estimating demand in all 80 low- and lower-middle-income countries, vaccinating year-round during the first ANC visit, in an attempt to try and generate peak demand estimates.

### Timing of supply

Although overall supply volumes are not likely to represent a barrier if low- and lower-middle-income countries start year-round maternal influenza immunization programs, the timing of supply may be an issue. A prior analysis of production cycles and shelf life duration demonstrated there are gap months during which neither Northern nor Southern Hemisphere formulation is available due to expiration dates of available vaccines and production cycles of forthcoming vaccines [12]. These gaps in vaccine availability can represent a challenge for countries that experience year-round circulation of the virus and hence opt for year-round vaccination, and for countries whose seasonality patterns require vaccination during availability gaps. To assess how many countries might be affected by such vaccine availability gaps, we examined seasonality information for each of the 54 Gavi countries alongside the aforementioned gap months. This information is presented in Table 1.

**Table 1:** Seasonality and potential gap in availability of Northern and Southern Hemisphere formulations.

		Potential availability gap SH					Potential availability gap NH					
	Jan	Feb	Mar	Apr	May	Jun	Jul	Aug	Sep	Oct	Nov	Dec
**Countries using NH formulation**												
*Afghanistan*	x	x	x									x
Burkina Faso		x	x									
*Burundi*		x	x	x	x	x						
Congo, DR	x	x	x	x	x							x
Côte d'Ivoire			x			x	x	x	x	x	x	
*Haiti*				x	x	x	x					
*Korea*, *DPR*	x	x	x	x								x
*Kyrgyzstan*	x	x	x									
Lao, PDR									x	x	x	
*Mauritania*	x	x										x
Niger	x	x										
Pakistan	x	x	x									x
*Papua New Guinea*	x	x	x	x							x	x
Rwanda		x	x	x	x	x						
Senegal									x	x	x	
*Solomon Islands*	x	x	x	x							x	x
*Sudan*	x										x	x
*Tajikistan*	x	x	x									
*Yemen*	x	x	x									x
**Countries using SH formulation**												
Bangladesh				x	x	x	x	x	x			
Cameroon						x			x	x	x	x
Central African Republic								x	x			
*Gambia*									x	x	x	
*Guinea*									x	x	x	
*Guinea-Bissau*									x	x	x	
India			x	x		x	x	x	x			
Kenya		x	x	x			x				x	
*Lesotho*					x	x	x	x	x			
Madagascar	x	x			x	x	x				x	
Nepal							x	x				
Nicaragua							x	x	x	x	x	
Sierra Leone								x		x		
Togo	x									x		x
Uganda				x	x			x	x	x	x	
*Zimbabwe*			x				x	x	x	x		
**Countries using both formulations**												
*Benin*	x	x	x			x			x	x	x	
Cambodia									x	x	x	x
*Chad*						x			x	x	x	x
*Comoros*	x	x			x	x	x				x	
*Djibouti*			x	x						x	x	
*Eritrea*			x	x						x	x	
Ethiopia			x	x						x	x	
Ghana			x	x		x	x			x	x	x
*Liberia*			x			x	x	x	x	x	x	
*Malawi*		x	x	x			x	x	x	x	x	
Mali	x	x	x						x	x		
*Mozambique*	x	x			x	x	x				x	
*Myanmar*		x					x	x	x	x		
Nigeria	x	x	x			x			x	x	x	
*Sao Tome and Principe*	x	x	x	x	x							x
*Somalia*			x	x						x	x	
*South Sudan*				x	x			x	x	x	x	
Tanzania		x		x								x
Zambia			x				x	x	x	x		

x Months with influenza virus circulation

Countries for which there is an incompatibility between the assumed period of vaccination and availability of supply

Countries in italics are countries for which seasonality data were extrapolated

A summary of results by number of countries is displayed in Table 2. Potential availability gaps affected countries both expected to use only one formulation as well as those alternating between the two formulations. Overall, about 60 percent of countries may face a supply timing issue, which would result in discontinuity of the immunization program, at a potentially critical time during or just before influenza activity.

**Table 2:** Countries eligible for New Vaccine Support (NVS) from Gavi in 2015 at risk of facing an availability gap of influenza vaccine formulations.

	#	# of countries at risk of facing an availability gap	% of countries at risk
Countries using SH formulation	16	6	38%
Countries using NH formulation	19	7	37%
Countries using both formulations	19	19	100%
Total	54	32	59%

## Discussion

In this article, we describe a model estimating the potential demand for seasonal influenza vaccines should maternal immunization programs be introduced in low- and lower-middle-income countries. The model integrates the latest recommendations that support integration of delivery of maternal influenza vaccination into antenatal care services. It also includes the latest data available regarding influenza seasonality in the tropics and subtropics, with extrapolations for countries where seasonality remains unknown. Furthermore, we assess vaccine demand in the context of both seasonal and year-round vaccination strategies, and we evaluate how delivery might be affected by a potential supply gap resulting from current vaccine production cycles. Results show that major drivers of demand, besides population and coverage assumptions, include year of introduction and scale-up assumptions for large countries.

Overall, our model shows that, assuming production capacity would remain at levels reported in 2015, global vaccine supply would be sufficient for maternal influenza immunization programs in low- and lower-middle-income countries. However, gaps in vaccine availability due to current production cycle-related processes and the limited shelf life of influenza vaccine might affect programs in several countries where demand corresponds to the supply gap period. Solutions to these barriers could include extending shelf life of seasonal influenza vaccines by three months as well as alternating between both hemisphere vaccine formulations [12]. Neither of these approaches has been put to practice in tropical or subtropical countries at the time of this publication.

### Model strengths and limitations

Our demand forecast model relies on several assumptions. Some of these assumptions are linked to the variability of how maternal immunization programs could be organized and for which parameters can be set (coverage, wastage, venue, time of vaccination, etc.). Other assumptions are linked to the lack of data. The most noticeable gap in data is regarding the seasonality of influenza in many low- and lower-middle-income countries, particularly in sub-Saharan Africa [35]. To fill this gap, the model includes extrapolation of seasonality information from neighbouring countries. Another limitation is the lack of information on determinants of availability and acceptance for the influenza vaccine in the focus countries and for the particular target group of pregnant women. While much has been written about this subject in high-income countries, vaccine acceptance by pregnant women in low- and lower-middle-income settings is still poorly characterized. If vaccine acceptance were decreased in low- and lower-middle-income countries, then our demand estimates would be high. The analysis aiming at assessing potential issues around manufacturing capacity and production cycles, it relies on aggressive adoption and coverage assumptions to better assess the strategy susceptibility to any global production limitations.

Finally, the model only looks at a particular risk group for severe influenza illness—pregnant women—which has been given priority by WHO, and is the only group under consideration for Gavi support [1]. Inclusion of other risk groups in our analyses, such as children under five years old, the elderly, health care workers, and individuals with underlying health conditions would provide better estimates of the overall potential market size for seasonal influenza vaccines in low- and lower-middle-income countries, should funding becomes available.

## Conclusion

We built a model to assess potential demand generated by the implementation of maternal influenza immunization programs in low- and lower-middle-income countries. Preliminary estimates from the base case and alternative scenarios analysed show that demand in 54 countries could grow from 7.7 to 16.0 million doses in 2020 to 27.0 to 61.7 million doses in 2029. Demand from these countries is characterized by a higher proportion of the Southern Hemisphere formulation.

Assuming current levels of production capacity remain unchanged, supply would not be constrained by additional demand generated by maternal influenza immunization programs, even under the most optimistic scenario tested in the study and considering all low- and lower-middle-income countries.

Although this additional demand would be located in regions where there is no or limited production capacity [33], the implementation of maternal influenza immunization programs in low- and lower-middle-income countries would maintain current levels of global readiness to respond to a future influenza pandemic.

## Supporting information

S1 Table Country groups and introduction dates considered in the analysis. (XLSX)

S2 Table Summary of model inputs, parameters and rationale. (DOCX)

S1 Fig Population figures smoothing method explanation. (DOCX)

S2 Fig Geographic distribution of demand estimates in 2029 (Base case scenario). (XLSX)
