# Supplementary material for: Forecasting demand for maternal influenza immunization in low- and lower-middle-income countries
Source: PLoS One. 2018 Jun 22;13(6):e0199470. doi: 10.1371/journal.pone.0199470 (PMC6014664; doi:10.1371/journal.pone.0199470)
Supplement: S2 Table — (DOCX) [file pone.0199470.s002.docx]

*S2 Table: Summary of model inputs, parameters and rationale*

| **Model inputs** | **Available parameters** | **Values used in the analysis** | **Rationale** |
| --- | --- | --- | --- |
| Countries | User selection  Income group  Gavi eligibility status  WHO region | Gavi eligible to New Vaccine Support in 2015  Low and lower-middle income countries | Gavi eligible countries were selected to provide information on potential size of a seasonal influenza vaccine support programme.  Low and lower middle income countries were selected as they are the initial focus of the project. |
| Introduction dates and scale up | Pre set  2020 early adopters  2022 medium adopters  2025 late adopters |  |  |
|  | Pre set  Large countries scale up coverage over a 3 year period  Small countries scale up in 1 year |  |  |
| Pregnant women population | User selection  Projection of live births  Low estimates  Medium estimates  High estimates | Medium estimates |  |
|  | Pre set  Addition of stillbirths |  |  |
| Duration of vaccination and vaccine formulation | Pre set  Number of peaks and months with influenza virus activity  Year-round influenza virus circulation = year-round vaccination  2 peaks = year-round vaccination  If 1 peak = duration of influenza season + 2 months |  |  |
|  | Pre set  Northern Hemisphere formulation  Southern Hemisphere formulation  Both formulations |  |  |
| Coverage | User selection  ANC attendance | ANC1+, ANC4+ | Base case scenario considers ANC4+ as repeated ANC visits increase the chance for pregnant women to be immunized.  An alternative scenario considers ANC1+, assuming influenza immunization would be given priority during the first ANC contact. |
|  | User selection  Multiplied by DTP1  Multiplied by DTP3 | DTP1 | DTP1 is used as a measure of country routine immunization performance and to reflect coverage of a single dose schedule vaccine. |
| Wastage | User selection  Open field | 5% | 5% wastage rate was chosen to reflect the use of a single dose vial product. |
| Buffer stock | User selection  Open field | 10% | Buffer stock was set at 10% to cover for unexpected interruptions in supply or sudden increase in demand. |
