# Supplementary material for: Forecasting demand for maternal influenza immunization in low- and lower-middle-income countries
Source: PLoS One. 2018 Jun 22;13(6):e0199470. doi: 10.1371/journal.pone.0199470 (PMC6014664; doi:10.1371/journal.pone.0199470)
Supplement: S1 Fig — (DOCX) [file pone.0199470.s003.docx]

*S1 Figure: Population figures smoothing method explanation*

United Nations population estimates are projected for periods of five years (e.g. 2020 to 2025).

|  | **2020 - 2025** | **2025 - 2030** |
| --- | --- | --- |
| UN Population Estimates available for 5-year period* | 5,000 | 7,500 |

**figures are only illustrative and not actual figures from the UN population estimates*

As these estimates would produce sharp changes in the demand for influenza vaccine in five year intervals, population data was smoothed to create a more uniform rate of change within each five year period. The year at the middle of the five year range was assumed to represent one-fifth of the number of births projected for that five-year period.

|  | **2020** | **2021** | **2022** | **2023** | **2024** | **2025** | **2026** | **2027** | **2028** | **2029** |
| --- | --- | --- | --- | --- | --- | --- | --- | --- | --- | --- |
| Smoothed Population | 800 | 900 | 1,000 | 1,100 | 1,200 | 1,300 | 1,400 | 1,500 | 1,600 | 1,700 |

A linear interpolation was then used to estimate the rate of change between two middle years—between 2022 and 2027, for example—and the number of births in the intervening years was modified while maintaining the projected number of births across each five year interval.
